# Study on the Impact of Collaborative Agglomeration of Manufacturing and Producer Services on PM_2.5_ Pollution: Evidence from Urban Agglomerations in the Middle Reaches of the Yangtze River in China

**DOI:** 10.3390/ijerph20043216

**Published:** 2023-02-12

**Authors:** Lei Gao, Jingran Zhang, Yu Tian, Xinyu Liu, Shuxin Guan, Yuhong Wu

**Affiliations:** 1 School of Economics and Management, Yanshan University, Qinhuangdao 066004, China; 2 School of Economics and Management, Beijing Forestry University, Beijing 100083, China; 3 Institute of Ancient Books, Jilin University, Changchun 130012, China

**Keywords:** industrial collaborative agglomeration, PM_2.5_ pollution, STIRPAT model, SDM model, urban agglomerations in the middle reaches of the Yangtze River

## Abstract

In this paper, using panel data of 28 cities in the middle reaches of the Yangtze River from 2003 to 2020 as the research sample, we built a dynamic spatial Durbin model based on the STIRPAT (stochastic impacts by regression on population, affluence, and technology) model and conducted an empirical study on the impact of the coordinated agglomeration of manufacturing and producer services on particulate matter (PM) 2.5 pollution. The results show a significant positive spatial spillover effect of PM_2.5_ pollution in the middle reaches of the Yangtze River. The coordinated agglomeration of manufacturing and producer services in the urban agglomerations there is conducive to reducing PM_2.5_ pollution. Similar to the inverted-U curve of the classic environmental Kuznets curve hypothesis, there is a significant inverted-U curve relationship between PM_2.5_ pollution and economic growth in urban agglomerations in the middle reaches of the Yangtze River. The proportion of coal consumption, the proportion of secondary industry, and the urbanization level are significantly and positively correlated with PM_2.5_ pollution in urban agglomerations in this area. Technological innovation, environmental regulation, and annual average humidity play an important role in addressing the PM_2.5_ pollution and spatial spillover effect. Industrial structure and technological innovation are the main ways for the coordinated agglomeration of manufacturing and producer services to affect PM_2.5_. The research conclusion can be of great practical significance to optimize the regional industrial layout, control PM_2.5_ pollution, and establish a sustainable development policy system in the middle reaches of the Yangtze River in China.

## 1. Introduction

With the rapid development of industrialization and urbanization, the urban air pollution problem is becoming increasingly serious, posing challenges to the human living environment and the sustainable development of society [1]. Particulate matter (PM) 2.5 is one of the most common air pollutants. PM_2.5_ has had profound effects on socioeconomics, the ecological environment, and human health [2]. Studies have shown that PM_2.5_ is toxic, causing respiratory diseases and even cancer [3,4]. The urban agglomeration in the middle reaches of the Yangtze River is the largest urban agglomeration in China and also one of the areas with a particularly serious haze pollution problem. According to the air quality index from 2015 to 2018 released by the Ministry of Environmental Protection in China, the average number of days in the middle reaches of the Yangtze River clusters was 247 days [5]. Therefore, research on PM_2.5_ pollution in the middle reaches of the Yangtze River is needed. This study will have a positive significance for promoting regional ecological environmental protection and realizing economic, societal, and ecological environment sustainability.

The environmental Kuznets curve (EKC) reveals that there is an inverted-U curve relationship between economic growth and environmental pollution in developed countries. This theory holds that after economic development reaches a certain extent, the role of economic growth on environmental quality will shift from inhibition to promotion [6]. From the perspective of economic development, the aggravation of environmental pollution is closely related to the extensive development mode, excessive proportion of coal consumption, lagging industrial structure, low environmental governance, and other factors [7]. The manufacturing industry is the core part of the industrial system that is dominated by the real economy, which effectively promotes the development of regional industrialization and urbanization [8]. The rapid development of the manufacturing industry is often accompanied by serious environmental pollution [9]. At present, China’s traditional manufacturing industry still has problems such as ecological environmental pollution, high resource energy consumption, and a lack of scientific and technological innovation capacity [10,11,12]. 

In recent years, with the transformation from the industrial economy to the service economy and knowledge economy as well as the refinement of the industrial division of labor, production services are gradually becoming separated from the manufacturing industry and have formed a phenomenon of synergistic agglomeration with the manufacturing industry. The coordinated agglomeration of the manufacturing industry and producer services is also considered an important factor affecting regional industrial upgrading, technological progress, energy conservation, and emissions reduction [13,14,15]. Industrial collaborative agglomeration refers to the spatial interdependence phenomenon among heterogeneous industries with a high correlation degree. This concept was first proposed by Ellison [16]. The generation mechanism of the collaborative agglomeration effect is mainly centered around the idea of Marshall space externalities, that is, the connection between intermediate inputs and end-product suppliers, sharing the labor market, increases information exchange and innovation opportunities [17,18]. Although scholars have carried out a lot of studies on the environmental effects of collaborative agglomeration, their conclusions are not consistent. On the one hand, industrial collaborative agglomeration may improve production efficiency and energy utilization efficiency through benign interaction between the external economy and industries [19,20], which can reduce regional economic growth while promoting environmental pollution [21,22,23]. On the other hand, industrial collaborative agglomeration may also further aggravate regional environmental pollution due to the crowding effect [24,25]. An industrial collaborative agglomeration is a special form in the process of the dynamic development of industrial agglomeration. With capital, talent, technology, and information as the carriers, it is the expression of the collaborative division of labor [26]. The existing research mainly measures the spatial co-agglomeration relationship between the manufacturing industry and producer services from the perspective of industrial agglomeration. The methods of industrial agglomeration measurement mainly include industry concentration [27], location entropy [28], the Huffender–Herschmann index [29], and the geographic concentration index [16]. Then, as the largest urban agglomeration in China, the coordinated agglomeration of manufacturing and producer services in the middle reaches of the Yangtze River has promoted local PM_2.5_ pollution or alleviated local PM_2.5_ pollution through its technical spillover effect. How to address local PM_2.5_ pollution in the Yangtze River Delta urban area needs to be explored in depth, and existing studies have not provided clear empirical evidence. 

The air pollution of urban agglomerations is characterized by seasonality, location, and industrial correlation [30,31]. Studies have shown that the emission, meteorological conditions, and topographic factors of pollution sources will have an important impact on air pollution in urban agglomerations [32,33]. At the same time, urbanization not only promotes regional economic development but also brings about environmental pollution problems [34]. The impact of industrial development on the environment will be affected by economic scale, environmental regulation, industrial structure, and production technology [35,36,37]. Therefore, after controlling for economic and natural factors, it is important to explore the impact of the collaborative agglomeration of manufacturing and producer services on PM_2.5_ pollution, which is of great practical significance in optimizing the regional industrial layout, controlling PM_2.5_ pollution, and establishing a policy system for sustainable development of the urban agglomerations in the middle reaches of the Yangtze River.

Based on this, this paper used panel data of 28 cities in the middle reaches of the Yangtze River from 2003 to 2020 as the research sample, constructed a dynamic spatial Durbin model based on the STIRPAT (stochastic impacts by regression on population, affluence, and technology) model, and conducted an empirical study on the impact of the collaborative agglomeration of manufacturing and producer services on PM_2.5_ pollution. There are three main innovations in this paper: (1) In terms of the research area, the urban agglomeration was taken as the research object, especially the urban agglomerations in the middle reaches of the Yangtze River, and the data of prefecture-level cities were used to capture the spatial effect of PM_2.5_ pollution in detail. (2) The joint inclusion of natural factors and economic factors as control variables in the model made the empirical results more reliable compared to traditional studies that focus only on economic variables. (3) Based on the lighting composite index, the urban agglomerations in the middle reaches of the Yangtze River can measure the urbanization level based on global nighttime light data to avoid the possible statistical error of the urbanization rate that only considers the representation of the urban population proportion.

The rest of this paper is organized as follows: Section 2 presents the theoretical analysis and research hypotheses; Section 3 presents the study area, theoretical models, and data sources; Section 5 presents the empirical results; Section 4 presents the discussion; and Section 6 presents the conclusions and policy implications.

## 2. Theoretical Analysis and Research Hypothesis

### 2.1. The Impact of Collaborative Agglomeration of Manufacturing and Producer Services on PM_2.5_ Pollution

The collaborative agglomeration effect among industries is mainly realized through externalities [16,17]. The collaborative agglomeration of the manufacturing industry and producer services can affect environmental pollution by deepening the division of labor, technological innovation, and spatial externalities [38]. Collaborative agglomeration makes the spillover effect of internal technologies more obvious, which can accelerate the research and development of clean energy technologies and promote the application of advanced technologies such as energy conservation and emission reduction in the manufacturing industry under the influence of technology. Collaborative agglomeration can enhance the effect of economies of scale, reduce the energy consumption per unit output through increasing the income from scale, and promote the centralized consumption of resources and the centralized treatment of pollutants so as to improve the efficiency of resource allocation. Collaborative agglomeration can improve the agglomeration level and professional capacity of producer services, while the agglomeration of producer services can provide more perfect production supporting services for local manufacturing enterprises, improve the service level of the manufacturing industry, and thus reduce the emission of air pollutants [39]. Based on this, we propose Hypothesis 1 (H1).

**H1:**  *The coordinated agglomeration of manufacturing industry and producer services can reduce the degree of PM_2.5_ pollution.*

### 2.2. The Collaborative Agglomeration of Manufacturing Industry and Producer Services and the Transmission Mechanism of PM_2.5_ Pollution

The coordinated agglomeration of the manufacturing industry and producer services can accelerate the interaction and integration between the two industries [19,20]. By increasing the input of human capital, technical capital, and other service factors, the structure of factor input and factor allocation are optimized, promoting the innovation of the production paradigm and then the green development of the industrial chain [36]. At the same time, the knowledge production and knowledge service functions of producer services make high-tech industries focus more on their core production links, and promote the traditional manufacturing industries associated with upstream and downstream industries to accelerate product upgrading and process improvement, and thus reduce the emission of air pollutants. Based on this, we propose Hypothesis 2 (H2).

**H2:**  *The coordinated agglomeration of manufacturing industry and producer services will reduce the degree of PM_2.5_ pollution by affecting the regional industrial structure.*

The collaborative agglomeration of industries enables the manufacturing industry to enjoy the intermediate input of high-added value and high-technology content provided by productive services more conveniently and improves the overall technological innovation level of the manufacturing industry through knowledge and technology spillover [40]. At the same time, the collaborative agglomeration of industries reduces the innovation cost of high-tech industries through face-to-face service, stimulates the innovation power of enterprises, and promotes innovative development [41]. Technological innovation can catalyze the scale effect and substitution effect of the agglomeration area, improve energy efficiency, and reduce pollution emissions [42]. Based on this, we propose Hypothesis 3 (H3).

**H3:**  *The coordinated agglomeration of manufacturing and producer services will reduce the degree of PM2.5 pollution by affecting the technology level.*

## 3. Materials and Methods

### 3.1. Study Area

**Figure 1:**
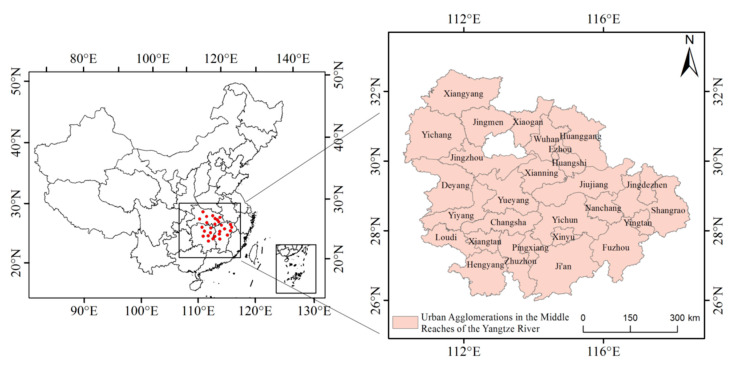
The urban agglomerations in the middle reaches of the Yangtze River.

The urban agglomeration in the middle reaches of the Yangtze River is the first superlarge national urban agglomeration recognized by the Chinese government. From the perspective of spatial location, the urban agglomeration in the middle reaches of the Yangtze River connects the east to the west and the south to the north (Figure 1). Urban agglomerations in the middle reaches of the Yangtze River plays an important role in China’s regional development pattern. Recent years have seen remarkable economic and social development in the urban agglomerations in the middle reaches of the Yangtze River, but environmental problems have become increasingly prominent [43,44]. The natural conditions and economic and social differences between the urban agglomeration in the middle reaches of the Yangtze River and that in the Beijing–Tianjin–Hebei region, the Yangtze River Delta, and the Pearl River Delta are mainly reflected in four aspects: (1) The urban agglomeration in the middle reaches of the Yangtze River is the largest urban agglomeration in China. It covers an area of 326,000 square kilometers, 1.5 times that of the Beijing–Tianjin–Hebei region and the Yangtze River Delta urban agglomeration, and six times that of the Pearl River Delta. (2) The urban agglomeration in the middle reaches of the Yangtze River has a developed water system, and its river distribution density is 0.557 km/km^2^, second only to the Yangtze River Delta urban agglomeration (0.78 km/km^2^) [45]. (3) The per capita GDP of the urban agglomeration in the middle reaches of the Yangtze River is relatively low. In 2021, the per capita GDP of the urban agglomeration in the middle reaches of the Yangtze River was CNY 73,800, much lower than that of the Beijing–Tianjin–Hebei urban agglomeration (CNY 117,300), the Yangtze River Delta urban agglomeration (CNY 123,500), and the Pearl River Delta urban agglomeration (CNY 153,000). (4) The urban agglomeration in the middle reaches of the Yangtze River is still in the middle stage of urbanization. In 2021, the urbanization rate of the urban agglomeration in the middle reaches of the Yangtze River was 61.75%, which lags behind the Beijing–Tianjin–Hebei urban agglomeration (65.8%), the Yangtze River Delta urban agglomeration (73.83%), and the Pearl River Delta urban agglomeration (80%). 

### 3.2. Model

The STIRPAT (stochastic impacts by regression on population, affluence, and technology) model originates from the IPAT (Impact = Population × Affluence × Technology) equation [46,47]. The expression for the IPAT equation is:

(1) I=P·A·T

where *I* represents environmental load; *P* represents population size; *A* represents affluence; and *T* represents the technical level.

York et al. constructed a STIRPAT model based on the IPAT equation [48], which is expressed as follows:

(2) $$I=aP^{b}A^{c}T^{d}\varepsilon$$

where *a* is a constant; *b*, *c*, and *d* are the index terms of *P*, *A*, and *T*, respectively; and ε is an error term. In Equation (2), *I*, *P*, *A*, and *T* have the same meaning as in Equation (1).

To eliminate possible heteroscedastic effects in model (2), all variables were extended [49]. The logarithmic-extended STIRPAT model is as follows (3):

(3) lnI=lna+blnP+clnA+dlnT+lnε

The advantage of the STIRPAT model is its scalability, and the explanatory variables allow it to add more relevant influencing factors to explore its impact on the environment [50]. To explore the effect of the collaborative agglomeration of manufacturing and producer services on PM_2.5_ pollution emission in the middle reaches of the Yangtze River in China, an extended STIRPAT model was constructed after logarithmic based on the study of Zhu [51], which is expressed as follows:

(4) $$lnPM_{2.5}=lna+b\ln P_{it}+c\ln A_{it}+d\ln T_{it}+e\ln ICA_{it}+h\ln X_{it}+\mu_{i}+\nu_{t}+\varepsilon_{it}$$

where *PM_2.5_* represents PM_2.5_ pollution; *ICA* represents the coordination and agglomeration of the manufacturing industry and producer services; *P* represents population density; *A* represents economic development level; *T* represents technical level; *X* represents the control variable group; *a* represents a constant and *b*, *c*, *d*, *e*, *f*, *g*, and *h* are index items; *i* represents the city; *t* represents the year; *u_i_* represents the fixed effect of city *i* that controls for features that do not change over time; *v_t_* represents the annual fixed effect, used to control the time-varying omitted variables and random shocks common to all cities; and $\varepsilon_{it}$ represents the error term.

This study comprehensively considers the spatial spillover (Appendix A provides a spatial autocorrelation test) and lag effects [52] in the presence of PM_2.5_ pollution. We constructed a dynamic space measurement model based on Equation (4). The specific model is as follows:

(5) $$\ln PM_{2.5it}=\tau \ln PM_{2.5it-1}+\rho \overset{N}{\underset{j=1}{\sum }}w_{ij}\ln PM_{2.5jt}+b\ln P_{it}+c\ln A_{it}+d\ln T_{it}+e\ln ICA_{it}+h\ln X_{it}+\mu_{i}+\nu_{t}+\varepsilon_{it}$$

where *w* represents the spatial weight matrix; *ρ* represents the spatial factor of the dependent variable; *τ* represents the dynamic factor of the dependent variable. In Equation (5), *PM_2.5_*, *P*, *A*, *T*, *ICA*, and *X* have the same meaning as in Equation (4). This study considers the endogenous problem of the economic distance matrix [53], so we adopted the inverse distance matrix as the spatial weight matrix. 

### 3.3. Variables Selection and Data Sources

#### 3.3.1. Variables Selection

In this study, PM_2.5_ concentration was used to measure PM_2.5_ pollution in urban agglomerations in the middle reaches of the Yangtze River. The core explanatory variable of this study is the co-agglomeration of the manufacturing industry and producer services, as measured by the industrial co-agglomeration index. Considering the research purpose and the availability of data in this paper, we should make reference to the idea of collaborative agglomeration among industries proposed by Ellison [16] and refer to the practice of Liu [54]. This paper first used the location entropy index to measure the agglomeration index of the manufacturing industry and producer service industry. Then, the cooperative agglomeration situation of the manufacturing industry and producer service industry was calculated based on the difference in the economic activity agglomeration index. The calculation formula is as follows:

(6) $$MAG_{i}=\frac{Q_{hi}/Q_{h}}{Q_{i}/Q}\;$$

(7) $$PAG_{i}=\frac{Q_{pi}/Q_{p}}{Q_{i}/Q}\;$$

(8) $$ICA_{ij}=\left(1-\frac{\left|MAG_{ij}-PAG_{ij}\right|}{MAG_{ij}+PAG_{ij}}\right)+\left|MAG_{ij}+PAG_{ij}\right|$$

where $ICA_{ij}$ represents the *j* year collaborative agglomeration index of the manufacturing industry and producer services in region *i*; $MAG_{ij}$ represents the location entropy index of the *j* year manufacturing industry in region *i*; $PAG_{ij}$ represents the location entropy index of the *j* year producer services industry in region *i*; $Q_{mij}$ and $Q_{pij}$ respectively indicate the number of employees in the manufacturing industry and producer service industry in *i* region in *j* year; $Q_{mj}$ and $Q_{pj}$ respectively indicate the employment figure in manufacturing and producer services in the *j* year; $Q_{ij}$ is the sum of the employment figure of manufacturing and producer services in *i* region in year *j*; and Q*_j_* is the sum of employment in manufacturing and producer services in China. The greater the value of $ICA_{ij}$, the higher the agglomeration degree of the two industries, and the more significant the synergism.

The control variables in this study included two parts: economic factors and natural factors. The economic factors were as follows: (1) Population density, characterized by the population per unit area [7], was expected to have a positive sign. (2) The economic development level was characterized by per capita GDP. According to the EKC hypothesis, both the primary and quadratic terms of per capita GDP were introduced into the model [55]. (3) Technical level, measured by the number of patents applied for in the current year, had a negative overdue limit. (4) Industrial structure, using the proportion of the added value of the secondary industry (including total industry and the construction industry), was measured by GDP. The process of industrialization leads to a large amount of fossil energy consumption and pollution emissions. At the same time, the rapid development of the construction industry has also increased the demand for highly energy-intensive products such as steel and cement [56,57]. The symbol was expected to be positive. (5) Energy structure was characterized by the proportion of coal consumption in the total energy consumption measurement [58]. The combustion of coal is an important source of PM_2.5_ pollution and was expected to be positive. (6) The level of opening up was measured by the total amount of foreign direct investment (FDI). Concerning the impact of FDI on environmental pollution, the academic community has formed two hypotheses: the pollution haven hypothesis and the pollution halo hypothesis. The former argues that FDI, through introducing a high-pollution industry in the host country, deteriorates the host country’s environmental quality, and the latter argues that FDI can introduce environmentally friendly products and technology to improve environmental quality [59]. Thus, the impact of FDI on PM_2.5_ pollution is uncertain. (7) To measure environmental regulation, the word frequency method was used to calculate environmental protection-related words in government reports to obtain the intensity coefficient of environmental regulation [60], and the expected symbol was negative. (8) The level of urbanization was characterized by the stable nighttime light data released by NOAA (National Oceanic and Atmospheric Administration) [61]. The urbanization process was accompanied by the agglomeration of population and factors, and the impact of the agglomeration process on environmental pollution is uncertain [62]. (9) Natural factors include average annual temperature, average annual relative humidity, and average annual wind speed [63,64].

#### 3.3.2. Data Sources

Based on the availability and completeness of the data, we built a complete panel dataset using data from 28 cities in the middle reaches of the Yangtze River during the period 2003–2020. Concentration data for PM_2.5_ were obtained from Washington University, St. Louis, MI, USA [65]. Stable light data were from the NOAA [66,67]. The data for the other variables in this paper were obtained from the China Urban Statistical Yearbook (2004–2021) [68], the Statistical Yearbook of Jiangxi Province (2004–2021) [69], the Statistical Yearbook of Hubei Province (2004–2021) [70], and the Statistical Yearbook of Hunan Province (2004–2021) [71]. The manufacturing industry studied in this paper mainly includes 13–43 categories classified in the Industry Classification of the National Economy (GB/T4754-2017) [72]. According to the Statistical Classification of Producer Services (2019), there are seven categories of producer services [73]. Table 1 shows the descriptive statistics of the variables. GDP is based on 2002 data adjusted using the GDP deflator, which is actual GDP excluding price changes.

## 4. Results

### 4.1. Inspection of the Spatial Measurement Models

According to the model setting, we used the spatial autoregressive model (SAR), the spatial error model (SEM), and the spatial Durbin model (SDM) as alternative models and analyzed them with Stata/SE16.0 (StataCorp, Lakeway, TX, USA). First, the appropriate spatial measurement model was selected via the Wald test and the likelihood-ratio test (Table 2). The results showed that the explanatory variable had a significant spatial spillover effect, indicating that the results rejected the null hypothesis, which showed that the SDM model cannot be simplified to the SAR model or the SEM model (Table 3). Therefore, the fixed-effect SDM model was used as the main tool for the empirical analysis.

### 4.2. Estimation Results for the Spatial Panel Durbin Model

The regression results in Table 4 show that the coordinated agglomeration of the manufacturing industry and producer services has a negative impact on PM_2.5_ pollution, which shows that the coordinated agglomeration of the manufacturing industry and producer services is conducive to alleviating PM_2.5_ pollution in the urban agglomerations in the middle reaches of the Yangtze River. The collaborative agglomeration of manufacturing industries and producer services may improve the local level of pollution by improving local production efficiency or environmental protection technology, or by spreading environmental awareness and sharing green management experience. With the classical EKC hypothesis that there is an inverted-U curve, the coefficient of the economic development index is positive and negative, and all data points are significant at the level of 1%, indicating that there is a significant inverted-U curve relationship between PM_2.5_ pollution and economic growth, namely that the degree of PM_2.5_ pollution increases with the economic growth level. Both industrial structure and energy structure have a significant positive impact on PM_2.5_ pollution, which is consistent with the conclusion of most studies on the relationship between industrial structure and environmental pollution [7]. This paper also found that the increased proportion of secondary industry will aggravate PM_2.5_ pollution. The increase in coal consumption has a significant effect on PM_2.5_ pollution, which is consistent with our expectations. The level of urbanization has a significant positive effect on PM_2.5_ pollution. It shows that the influence of urbanization levels on PM_2.5_ pollution is mainly reflected in the scale effect. This is because the urban agglomerations in the middle reaches of the Yangtze River are at a relatively early stage of urbanization. Urbanization has generated a large amount of demand for infrastructure construction, which has driven the excessive growth of the heavy chemical industry with high energy consumption and high emissions characteristics, such as cement and steel, and caused a large amount of energy consumption and environmental pollution [62]. FDI has a significant positive effect on PM_2.5_ pollution. It shows that the technology spillover effect brought by FDI to the middle reaches of the Yangtze River is not obvious. The coefficient of environmental regulation intensity and technical innovation is significantly negative, indicating that government environmental regulation effectively suppresses PM_2.5_ pollution. Among the natural factor control variables, annual mean humidity has a significant negative effect on PM_2.5_ pollution, probably because increased air humidity helps inhibit aerosol formation and thus reduces PM_2.5_ pollution; population density and other natural indicators were not significant.

### 4.3. Decomposition of Direct and Indirect Effects

Referring to the study of LeSage and Pace, we further decomposed the effects of each factor on PM_2.5_ pollution into direct effects and indirect effects [74]. Because this paper uses a dynamic spatial panel data model, we discuss empirical results for the long-term effects of time lag effects.

Observation Table 5 shows that the direct effect of the control variables on PM_2.5_ contamination in this region is in line with the aforementioned analysis of the regression estimation results. Both the direct and indirect effects of the collaborative agglomeration of the manufacturing industry and producer services are significantly negative, indicating that the coordinated agglomeration of the manufacturing industry and producer services in the urban agglomerations in the middle reaches of the Yangtze River has an effect on reducing the PM_2.5_ pollution in the surrounding areas. This result proves that Hypothesis 1 is correct. The possible explanation is that, on the one hand, the employment opportunities brought by the coordinated agglomeration of the manufacturing industry and producer services contribute to population migration from the surrounding underdeveloped areas to the developed areas, and the environmental pollution scale effect of population agglomeration in these areas is weakened. On the other hand, under the pressure of performance assessment or promotion, local governments have the imitation effect of industrial green development. The direct and indirect effects of economic development have an inverted-U curve relationship, and they are significant at the 1% level. This result shows that in the early stage of economic development, the difficulty of PM_2.5_ pollution control in the middle reaches of the Yangtze River and its surrounding areas will intensify. However, as the level of economic development continues to rise, the PM_2.5_ pollution in this region and its surrounding areas will decrease. The reason for this situation may be that economic improvement will drive improvements in energy-saving technology and pollution control technology. Both the direct and indirect effects of energy structure and industrial structure are significantly positive. This shows that the increase in the proportion of coal consumption in the urban agglomeration of the middle reaches of the Yangtze River is not conducive to the improvement in PM_2.5_ pollution in the surrounding areas. The positive relationship between the degree of urbanization and PM_2.5_ concentration indicates that the current urbanization pattern will still increase the pollution level of PM_2.5_ in the middle reaches of the Yangtze River in the long term. Both the direct and indirect effects of FDI were significantly positive, consistent with the pollution haven hypothesis. Finally, both the direct and indirect effects of technological innovation and environmental regulation are significantly negative, which is conducive to PM_2.5_ pollution control in the surrounding cities.

In the natural factor control variables, the direct and indirect effects of annual average humidity were negative and significant. The results prove that the climatic characteristics of urban agglomerations in the middle reaches of the Yangtze River play an important role in the diffusion of PM_2.5_ pollution. However, the direct and indirect effects of population density and other natural factors were not significant.

### 4.4. Robustness Test

Considering the possible extreme values of the variables in this paper, this study used the robustness test of the model by shrinking the data. Tail reduction for all variables at the 5% and 95% levels was re-estimated using an SDM model with spatial fixed effects. According to Table 6 and Table 7, the overall estimation results of the model tended to be the same as the aforementioned benchmark regression results. Although the estimation coefficient of individual variables was slightly different, the fundamental change in direction and significance and the fitting degree of significance were good, which shows that the empirical estimation results of the selected model have good robustness.

### 4.5. Analysis of the Influence Path of the Manufacturing and Producer Services Collaborative Agglomeration on PM_2.5_ Pollution

According to the analysis above, the coordinated agglomeration of the manufacturing industry and producer services in the middle reaches of the Yangtze River is conducive to alleviating PM_2.5_ pollution. To further understand the transmission path of the coordinated agglomeration of manufacturing and producer services, we referred to the method of Baron and Kenny [75], selected the industrial structure and technological innovation as the intermediary variables, and identified the above conduction pathway with the help of the intermediary effect model.

According to Table 8, industrial structure and technological innovation play a partial intermediary role in the influence mechanism of the coordinated agglomeration of manufacturing and producer services on PM_2.5_, which shows that industrial structure and technological innovation are the main ways for the coordinated agglomeration of manufacturing and producer services in the middle reaches of the Yangtze River to affect PM_2.5_ pollution. Specifically, when the industrial structure is regarded as an intermediary variable, the estimated coefficient of collaborative agglomeration of manufacturing and producer services is significant at the level of 1%, indicating that the industrial structure of urban agglomerations in the middle reaches of the Yangtze River has some intermediary effect on the influence mechanism of the coordinated agglomeration of manufacturing and producer services on PM_2.5_. This also means that the coordinated agglomeration of manufacturing and producer services can significantly optimize the local industrial structure, thus reducing the PM_2.5_ pollution level. Similarly, when technological innovation is regarded as an intermediary variable, the estimated coefficient of collaborative agglomeration of manufacturing and producer services is significant at the level of 10%, indicating that technological innovation in urban agglomerations in the middle reaches of the Yangtze River has some intermediary effect on the influence mechanism of collaborative agglomeration of manufacturing and producer services on PM_2.5_. It can be seen that the impact of the coordinated agglomeration of manufacturing and producer services in the middle reaches of the Yangtze River on PM_2.5_ pollution is realized through the industrial structure and technological innovation. The above results prove that Hypotheses 2 and 3 are correct.

## 5. Discussion

The urban cluster in the middle reaches of the Yangtze River, one of the first superlarge national urban clusters approved by the Chinese government, is one of the most polluted areas with PM_2.5_ in China. Therefore, in the context of high-quality economic and ecological development, it is necessary to explore the impact of the synergistic agglomeration of manufacturing and producer services on PM_2.5_ pollution, while considering both economic and natural factors. The empirical results of this study show that there is a significant positive spatial spillover effect of PM_2.5_ pollution in the urban agglomerations in the middle reaches of the Yangtze River, and the coordinated agglomeration of manufacturing and producer services is conducive to alleviating PM_2.5_ pollution there. The above results support Ellison, Tang, and Wu, who state that industrial collaborative agglomeration may improve production efficiency and energy utilization efficiency through the external economy and benign interaction between industries [17,18,19,20]. Clusters can also be understood as the middle reaches of the Yangtze River urban agglomeration manufacturing, and producer services can enhance the local effect of economies of scale by increasing scale income to reduce energy consumption per unit output, promoting the concentration of resource consumption and centralized management of pollutants, building a circular economic system, improving the efficiency of resource allocation, and manufacturing green development.

In addition to the above results, we also demonstrated the inverted-U curve relationship between PM_2.5_ pollution and economic growth in the middle reaches of the Yangtze River, which is consistent with the EKC hypothesis [6,76]. In addition, urbanization, the proportion of secondary industry, and the coal consumption structure will all have a significant positive impact on PM_2.5_ pollution in urban agglomerations in the middle reaches of the Yangtze River. Compared with social and economic factors, natural factors play a more stable role in the influence of the manufacturing industry and producer service industry in the urban agglomerations in the middle reaches of the Yangtze River.

## 6. Conclusions

In this paper, taking panel data of 28 cities in the middle reaches of the Yangtze River from 2003 to 2020 as the research sample, we built a dynamic spatial Durbin model based on the STIRPAT model and conducted an empirical study on the impact of the coordinated agglomeration of manufacturing and producer services on PM_2.5_ pollution. The results show that there is a significant positive spatial spillover effect of PM_2.5_ pollution in the middle reaches of the Yangtze River. The coordinated agglomeration of manufacturing and producer services in the urban agglomerations in the middle reaches of the Yangtze River is conducive to reducing PM_2.5_ pollution. Following the classic EKC hypothesis, there is a significant inverted-U curve relationship between PM_2.5_ pollution and economic growth in urban agglomerations in the middle reaches of the Yangtze River. The proportion of coal consumption, the proportion of secondary industry, and the degree of urbanization are significantly and positively correlated with PM_2.5_ pollution in urban agglomerations in the middle reaches of the Yangtze River. Technological innovation, environmental regulation, and annual average humidity play an important role in suppressing PM_2.5_ pollution and the spatial spillover effect in the urban agglomerations in the middle reaches of the Yangtze River. Industrial structure and technological innovation are the main ways for the coordinated agglomeration of manufacturing and producer services in the middle reaches of the Yangtze River to have an impact on PM_2.5_ pollution. This conclusion can be of great practical significance in optimizing the regional industrial layout, controlling PM_2.5_ pollution, and establishing a sustainable development policy system in the middle reaches of the Yangtze River. Based on the above conclusions, the recommendations of this paper mainly focus on the following three aspects.

(1) Based on the advantageous industries of the urban agglomerations in the middle reaches of the Yangtze River, the layout of the manufacturing industry and producer service industry is scientifically coordinated. We will continue to optimize the internal structure of industries and appropriately control the development scale of resource-dependent industries to narrow the gap between the industrial collaborative agglomeration level within urban agglomerations, give full play to the knowledge of the spillover effect between manufacturing and producer services in different cities, and reduce the PM_2.5_ pollution degree in urban agglomerations in the middle reaches of the Yangtze River. (2) We will adjust the energy consumption structure of urban agglomerations in the middle reaches of the Yangtze River and reduce the proportion of coal in the energy consumption structure. The proportion of clean energy used should be increased by adjusting the energy structure. At the same time, we will further strengthen the implementation of a series of policies, such as setting emission reduction targets and lowering emissions limits, giving full play to the guiding role of government funds, and encouraging highly polluting enterprises to adopt technological transformation. We will strengthen the rectification and management of industries with high emissions and high pollution and eliminate the illegal discharge of pollutants. We will change the economic development mode and force the green upgrading of industrial structure and energy structure through market environmental regulation. (3) We will establish a joint PM_2.5_ pollution prevention and control mechanism in urban agglomerations in the middle reaches of the Yangtze River. While strengthening the economic and cultural cooperation within urban agglomerations, the cooperation between local governments in air pollution control should also be strengthened. Cross-regional air pollution prevention and control requires cooperation between governments, and only joint governance can achieve real results.

The limitations of this study are as follows: PM_2.5_ contamination is the result of the interaction between human activities and natural factors. The degree of PM_2.5_ pollution is not only related to the energy structure, FDI, urbanization level, industrial structure characteristics, and other factors; the structure of industrial collaborative agglomeration, industrial distribution, and residents’ lifestyles also affect regional PM_2.5_ pollution. Our future research will combine the data of subdivided industries, monthly meteorological data, and animal and plant data in the middle reaches of the Yangtze River to discuss the role of the above factors in the coordinated agglomeration of manufacturing and producer services on PM_2.5_ pollution and build a more reasonable index system. In addition, we will carry out a study on the differentiation between provinces and cities within the urban agglomeration in the middle reaches of the Yangtze River from the perspective of regional heterogeneity.

## Figures and Tables

**Table 1:** Variables in the empirical model.

Variable	Meaning	Obs	Mean	Std. Dev.	Min	Max
*PM_2.5_*	Concentration of PM_2.5_ (μg/m^3^)	504	47.269	10.556	26.289	72.446
*ICA*	Industrial synergy and agglomeration index	504	2.786	0.158	2.354	2.998
*P*	Population density (million people/km^2^)	504	438.732	184.479	87.090	1434.722
*A*	Per capita GDP (CNY ten thousand)	504	5.6583	0.734	0.3357	13.1440
*T*	Number of patents (pieces)	504	2478.294	75,296.12	17	695,714
*ES*	Coal consumption structure (%)	504	79.135	15.439	23.426	95.988
*FDI*	Foreign investment amount (USD ten thousand)	504	71,243.831	137,652.71	1095	1,230,900
*IS*	The proportion of secondary industry (%)	504	48.847	7.673	30.660	64.320
*REG*	Environmental regulation	504	0.005	0.003	0.000	0.013
*URB*	Urbanization level	504	10.21	10.63	0.01	57.81
*AT*	Average temperature (°C)	504	17.457	0.846	15.689	19.428
*ARH*	Average relative humidity (%)	504	76.284	2.733	70.738	82.321
*AWP*	Average wind speed (m/s)	504	4.362	0.775	2.983	6.974

**Table 2:** Tests of Wald and LR.

Type of Inspection	Null Hypothesis	Statistic	*p*-Value	Results
Wald test	SEM excel SDM	74.10	0.000	Rejected
	SAR excel SDM	96.75	0.000	Rejected
LR test	SEM excel SDM	89.79	0.000	Rejected
	SAR excel SDM	70.09	0.000	Rejected

**Table 3:** Tests of Hausman.

Type of Inspection	Statistic	*p*-Value	Results
Hausman	12.84	0.025	Rejected
LR test (Comparison of double fixation versus individual fixation)	68.77	0.000	Rejected
LR test (Comparison of double fixation versus temporal fixation)	601.77	0.000	Rejected

**Table 4:** Regression results for the SDM.

Variable	Value	Variable	Value
*lnICA*	−2.0447 **	W*lnICA*	−10.4508 **
	(−2.3991)		(−2.1220)
*lnP*	−11.3581	W*lnP*	8.4267
	(−1.8896)		(0.3954)
*lnA*	1.9241 **	W*lnA*	0.6554
	(0.3796)		(0.4927)
*lnA^2^*	−2.2357 **	W*lnA*	−0.5053
	(−2.1984)		(−0.2967)
*lnT*	−0.2629 ***	W*lnT*	−0.1994 **
	(0.7301)		(−0.3726)
*lnES*	0.0235 **	W*lnES*	0.0161 **
	(2.0266)		(0.4568)
*lnFDI*	0.5000 **	W*lnFDI*	3.4229 ***
	(2.0361)		(2.7393)
*lnIS*	0.0535 **	W*lnIS*	0.0959 *
	(2.0806)		(1.6172)
*lnREG*	−40.1717 **	W*lnREG*	−2.0 × 10^2^ **
	(−0.7213)		(−1.0628)
*lnURB*	0.0876 ***	W*lnURB*	0.0184 ***
	(0.0237)		(0.0713)
*lnAT*	0.2988	W*lnAT*	−0.2721
	(0.8258)		(−0.4421)
*lnARH*	−0.2144 **	W*lnARH*	−0.1884 **
	(−2.4711)		(−1.4410)
*lnAWP*	−0.7844	W*lnAWP*	−1.9490
	(−1.4953)		(−1.5537)
*ρ*	0.8088 ***		
	(34.206)		
*N*	504		
R-squared	0.7998		

Note: *** *p* < 0.01, ** *p* < 0.05, * *p* < 0.1. Standard errors are in parentheses.

**Table 5:** Decomposition of effects in the dynamic SDM model.

Variable	LR_Direct	LR_Indirect	LR_Total
*lnICA*	−5.4939 ***	−1.0 × 10^2^ **	−1.1 × 10^2^ **
	(−2.6206)	(−1.9982)	(−2.0299)
*lnP*	−11.0794	−7.4557	−18.5351
	(−1.1474)	(−0.0406)	(−0.0967)
*lnA*	0.0867 ***	0.6184 ***	0.7160 ***
	(0.0265)	(0.2525)	(0.2768)
*lnA^2^*	−2.8476 ***	−20.2261 *	−23.0737 *
	(−2.7138)	(−1.7175)	(−1.9001)
*lnT*	−0.2557 ***	−0.1638 **	−0.4195 ***
	(0.6886)	(0.0423)	(0.1043)
*lnES*	0.0340 **	0.3020 *	0.3360 *
	(2.1911)	(1.1078)	(1.1846)
*lnFDI*	1.5944 ***	31.8500 ***	33.4444 ***
	(3.2237)	(2.7658)	(2.7997)
*lnIS*	0.0934 ***	1.2239 **	1.3173 **
	(2.9724)	(2.0811)	(2.1658)
*lnREG*	−4.7166 **	−1.3 × 10^3^ **	−1.3 × 10^3^ *
	(−0.0556)	(−0.8192)	(−0.7894)
*lnURB*	0.1247 ***	0.3536 ***	0.7434 ***
	(0.0253)	(0.1470)	(0.2133)
*lnAT*	0.3326	0.4122	0.7448
	(0.8913)	(0.0956)	(0.1679)
*lnARH*	−0.3215 ***	−3.0989 ***	−3.4204 ***
	(−3.5520)	(−3.7921)	(−4.0748)
*lnAWP*	−1.5722	−22.4385	−24.0107
	(−2.9798)	(−2.3498)	(−2.4594)
*ρ*	0.8688 ***		
	(30.6924)		
*N*	504		
R-squared	0.7727		

Note: *** *p* < 0.01, ** *p* < 0.05, * *p* < 0.1. Standard errors are in parentheses.

**Table 6:** Robustness test.

Variable	Value	Variable	Value
*lnICA*	−1.9642 **	W*lnICA*	−10.7764 **
	(−2.1649)		(−1.9760)
*lnP*	−43.1141	W*lnP*	−38.4443
	(−1.8718)		(−0.3575)
*lnA*	1.5740 **	W*lnA*	2.3235 *
	(2.0194)		(1.3310)
*lnA^2^*	−1.6940 **	W*lnA*	−2.4435 **
	(−2.0194)		(−1.4310)
*lnT*	0.6158 **	W*lnT*	−0.2160 ***
	(2.0812)		(−0.3996)
*lnES*	0.0272 **	W*lnES*	0.0301
	(2.0740)		(0.8289)
*lnFDI*	0.5909 **	W*lnFDI*	4.5868 ***
	(2.2153)		(3.4299)
*lnIS*	0.0288	W*lnIS*	0.1606 **
	(1.0529)		(2.4809)
*lnREG*	−33.9690 **	W*lnREG*	−1.6 × 10^2^ **
	(−0.5411)		(−0.7618)
*lnURB*	0.0687 ***	W*lnURB*	0.0527 ***
	(0.0244)		(0.0237)
*lnAT*	0.2863	W*lnAT*	−0.0598
	(0.7528)		(−0.0926)
*lnARH*	−0.2945 ***	W*lnARH*	−0.1535 ***
	(−3.2159)		(−1.1268)
*lnAWP*	−0.3857	W*lnAWP*	−4.0156
	(−0.7480)		(−3.1371)
*ρ*	0.8688 ***		
	(30.6924)		
*N*	504		
R-squared	0.7727		

Note: *** *p* < 0.01, ** *p* < 0.05, * *p* < 0.1. Standard errors are in parentheses.

**Table 7:** Spatial effect decomposition results for the robustness test.

Variable	LR_Direct	LR_Indirect	LR_Total
*lnICA*	−5.1085 **	−92.7059 *	−97.8145 *
	(−2.3921)	(−1.8364)	(−1.8659)
*lnP*	−59.5217	−5.4 × 10^2^	−6.0 × 10^2^
	(−1.3578)	(−0.6146)	(−0.6535)
*lnA*	2.5017 ***	29.2736 **	29.9121 **
	(2.7406)	(2.2416)	(2.4527)
*lnA^2^*	−2.6215 ***	−29.2806 **	−31.9021 **
	(−2.8526)	(−2.3517)	(−2.4760)
*lnT*	0.6830 **	2.2925	2.9755
	(2.1059)	(0.5625)	(0.7016)
*lnES*	0.0411 ***	0.4027 *	0.4438 *
	(2.5806)	(1.6582)	(1.7549)
*lnFDI*	1.9014 ***	38.3937 ***	40.2952 ***
	(3.6880)	(3.2568)	(3.2944)
*lnIS*	0.0758 **	1.4453 **	1.5211 **
	(2.2762)	(2.3263)	(2.3675)
*lnREG*	−2.8825 **	−9.6 × 10^2^ **	−9.5 × 10^2^ **
	(−0.0322)	(−0.5911)	(−0.5653)
*lnURB*	0.1246 ***	0.2571 ***	0.7933 ***
	(0.0277)	(0.2140)	(0.2079)
*lnAT*	0.3723	1.9485	2.3208
	(0.9631)	(0.4646)	(0.5386)
*lnARH*	−0.3997 ***	−3.0611 ***	−3.4608 ***
	(−4.2096)	(−3.8811)	(−4.2827)
*lnAWP*	−1.5498 ***	−33.4868 ***	−35.0366 ***
	(−2.6906)	(−3.1813)	(−3.2284)
*ρ*	0.8688 ***		
	(30.6924)		
*N*	504		
R-squared	0.7727		

Note: *** *p* < 0.01, ** *p* < 0.05, * *p* < 0.1. Standard errors are in parentheses.

**Table 8:** Empirical results of the influence path of the coordinated agglomeration of manufacturing industry and producer services on PM_2.5_ pollution.

	*lnIS*	*lnPM_2.5_*	*lnT*	*lnPM_2.5_*
*lnICA*	0.0115 ***	−0.0462 **	−0.0178 **	−0.0384 **
	(0.1725)	(0.2941)	(0.0668)	(0.1941)
*lnP*	0.0784	−0.6264	0.1362 **	−0.4455
	(0.0181)	(0.0274)	(0.0071)	(0.0211)
*lnA*	2.0927	3.5862 ***	−4.6822 ***	4.6816 ***
	(1.4267)	(1.7924)	(0.4815)	(1.5832)
*lnA^2^*	−0.0854	−0.1892 **	0.1658 ***	−0.1778 ***
	(0.0529)	(0.1672)	(0.0178)	(0.0568)
*lnT*	−0.0315 **	−0.0827 **		−0.0727 **
	(0.0131)	(0.0592)		(0.0687)
*lnES*	−0.0024	0.1142 **	−0.0315 **	0.1061 *
	(0.1581)	(0.1805)	(0.0131)	(0.1794)
*lnFDI*	−0.0634	0.0402 **	−0.0206	0.0315 **
	(0.0548)	(0.0331)	(0.0202)	(0.0131)
*lnIS*		0.1629 *	−0.0454	0.1578 *
		(0.0892)	(0.0327)	(0.0952)
*lnREG*	−0.1744 ***	−0.0461 ***	−0.0315 **	−0.0362 **
	(0.0318)	(0.0472)	(0.0131)	(0.0374)
*lnURB*	−0.1613 **	0.0391 **	0.0182	0.0472 *
	(0.0678)	(0.0862)	(0.0271)	(0.0781)
*lnAT*	−2.2711 *	0.1136	1.0194 **	0.1034
	(1.2374)	(1.3751)	(0.4848)	(1.4051)
*lnARH*	−0.8354	−0.2812 **	0.3533 *	−0.2713 **
	(0.5394)	(0.5217)	(0.2112)	(0.6012)
*lnAWP*	0.1969	−0.0479	−0.0585	−0.0698
	(0.2261)	(0.1375)	(0.0874)	(0.2571)
*ρ*	−219.1298 *	−157.9166	447.1221 ***	−147.9175
	(125.0421)	(127.2772)	(81.2206)	(128.3871)
*N*	504	504	504	504
R-squared	0.1388	0.0613	0.0812	0.0503

Note: *** *p* < 0.01, ** *p* < 0.05, * *p* < 0.1. Standard errors in parentheses.

## Appendix A

This paper uses the Moran’s *I* index [77] to test whether PM_2.5_ pollution exists in the urban agglomeration in the middle reaches of the Yangtze River. The calculation formula is as follows:

(A1) $$I=\frac{n\sum_{i=1}^{n}\sum_{j=1}^{n}w_{ij}\left(x_{i}-\bar{x}\right)\left(x_{j}-\bar{x}\right)}{\sum_{i=1}^{n}\sum_{j=1}^{n}w_{ij}\sum_{i=1}^{n}\left(x_{i}-\bar{x}\right)}$$

where *I* represents the Moran’s *I* index; *n* represents the 28 prefecture-level cities within the urban agglomeration in the middle reaches of the Yangtze River; $x_{i}$ and $\;x_{j}$ represent the concentration values of PM_2.5_ contamination in cities *i* and *j*, respectively; $\bar{x}$ represents the mean value of the PM_2.5_ concentration across all cities; and *w_ij_* represents the spatial weight matrix. The Moran’s *I* statistic is valued at [–1, 1], and when the Moran’s *I* symbol is positive (negative), it indicates that the data has a spatial positive (negative) correlation. The value of the Moran’s *I* dataset used for the analysis is directly proportional (inversely proportional) to the spatial aggregation degree, and the greater the absolute value of this value, the more obvious the spatial correlation is.

The results of the spatial correlation test (Table A1) showed that the Moran’s *I* index was positive under the inverse distance matrix, and they all passed the significance level test of 1%. The above results indicate a significant positive spatial spillover effect of PM_2.5_ pollution in the urban agglomeration in the middle reaches of the Yangtze River. In other words, the local PM_2.5_ pollution is affected by the neighboring areas, which is specifically characterized by the spatial distribution of “high-high” and “low-low”.

**Table A1:** Moran’s *I* of PM_2.5_ pollution in the urban agglomerations in the middle reaches of the Yangtze River based on geographical distance weight matrix.

Year	Moran’s *I*	E(I)	sd(I)	z	*p*-Value
2003	0.176	−0.037	0.033	6.503	0.000
2004	0.176	−0.037	0.033	6.505	0.000
2005	0.212	−0.037	0.033	7.536	0.000
2006	0.184	−0.037	0.033	6.711	0.000
2007	0.205	−0.037	0.033	7.352	0.000
2008	0.214	−0.037	0.033	7.602	0.000
2009	0.208	−0.037	0.033	7.491	0.000
2010	0.217	−0.037	0.033	7.724	0.000
2011	0.231	−0.037	0.033	8.123	0.000
2012	0.223	−0.037	0.033	7.920	0.000
2013	0.230	−0.037	0.033	8.102	0.000
2014	0.180	−0.037	0.033	6.634	0.000
2015	0.226	−0.037	0.033	7.994	0.000
2016	0.192	−0.037	0.033	7.016	0.000
2017	0.148	−0.037	0.033	5.670	0.000
2018	0.202	−0.037	0.033	7.292	0.000
2019	0.209	−0.037	0.033	7.472	0.000
2020	0.164	−0.037	0.033	6.123	0.000

## References

[1] Zhang Z., Zhang G., Su B. (2022). The Spatial Impacts of Air Pollution and Socio-Economic Status on Public Health: Empirical Evidence from China. Socio-Econ. Plan. Sci..

[2] Guo X., Wang Y., Mei S., Shi C., Liu Y., Pan L., Li K., Zhang B., Wang J., Zhong Z. (2022). Monitoring and modelling of PM_2.5_ concentration at subway station construction based on IoT and LSTM algorithm optimization. J. Clean. Prod..

[3] Yue H., He C., Huang Q. (2020). Stronger policy required to substantially reduce deaths from PM_2.5_ pollution in China. Nat. Commun..

[4] Chen M., Huang B., Liu Y. (2022). Effects of PM_2.5_ concentration on mortality in China: A study based on city-level panel data. Prog. Geogr..

[5] Chen M., Wang S., Yue H., Zhang X. (2021). Measurement and Analysis of Spatial Correlation of Haze Pollution in Urban Agglomerations in the Middle Reaches of the Yangtze River. East China Econ. Manag..

[6] Grossman G.M., Krueger A.B. (1992). Environmental impacts of a north american free trade agreement. Natl. Bur. Econ. Res. Work. Pap..

[7] Shao S., Li X., Cao J., Yang L. (2016). China’s economic policy choices for governing smog pollution based on spatial spillover effects. Econ. Res. J..

[8] Deng Z., Huang Q. (2021). On the Impact of Manufacturing Agglomeration on Pollution in Western China from the Perspective of High-quality Development. Inq. Into Econ. Issues.

[9] Jin H., Liu C., Chen S. (2022). Why is COD pollution from Chinese manufacturing declining?: The role of environmental regulation. J. Clean. Prod..

[10] An S. (2018). A Research on the Path of Promoting High-quality Economic Development: A Literature Review. Contemp. Econ. Manag..

[11] Song X., Li J. (2022). Industrial Collaborative Agglomeration, Local Government Competition and Green Development of Manufacturing Industry. Econ. Surv..

[12] Sun L., Zhang J., Xu N. (2022). Effect Evaluation and Mechanism Research on Enhancing Enterprise Green Total Factor Productivity of “Diversified”Industrial Synergistic Agglomeration. Inq. Into Econ. Issues.

[13] Chen J., Liu Y., Chen H. (2016). Market Potential, Industrial Co-agglomeration and Regional Wages Income: Empirical Evidence from 151 Chinese Cities. Nankai J..

[14] Cai H., Xu Y. (2018). Co-agglomeratio, trade openness and haze pollution. China Popul. Resour. Environ..

[15] Yuan Y., Gao K. (2020). The synergetic agglomeration of industries, spatial knowledge spillovers and regional innovation efficiency. Stud. Sci. Sci..

[16] Ellison G., Glaeser E.L. (1997). Geographic concentration in U.S. manufacturing industries: A dartboard approach. J. Political Econ..

[17] Ellison G., Glaeser E.L., Kerr W.R. (2010). What causes industry agglomeration? evidence from coagglomeration patterns. Am. Econ. Rev..

[18] Wang H., Zhou Y. (2022). Industrial Agglomeration and Urban Industrial Carbon Emissions: Evidence from Economic Development Zones. Soft Sci..

[19] Tang X., Zhang X., Li Y. (2018). The Effect of Coordinated Development between Manufacturing Industry and Producer Services. J. Quant. Technol..

[20] Wu X. (2018). Have Producer Services and Manufacturing Industry Co-agglomeration Promoted Total Factor Productivity. Collect. Essays Financ. Econ..

[21] Lu F., Yang H. (2020). Industrial Co-agglomeration and Environmental Pollution Control: Impetus or Resistance. J. Guangdong Univ. Financ. Econ..

[22] Guo W., Huang F. (2020). How does the co-agglomeration of high-tech industries and producer services affect the quality of economic growth?. Ind. Econ. Res..

[23] Tang C., Qiu J., Zhang L., Li H. (2021). Spatial Econometric Analysis on the Influence of Elements Flow and Industrial Collaborative Agglomeration on Regional Economic Growth: Based on Manufacturing and Producer Services. Econ. Geogr..

[24] Yu B., Yang H., Jin G. (2015). Can industrial agglomeration improve regional economic efficiency? Spatial Measurement Analysis based on Chinese urban data. J. Zhongnan Univ. Econ. Law.

[25] Wang Y., Sun C. (2019). Influence of Industrial Co-agglomeration on Industrial Structure Optimization: Based on The Empirical Analysis of High-tech Industry and Producer services. Inq. Into Econ. Issues.

[26] Zhang H., Han A., Yang Q. (2017). Spatial Effect Analysis of Synergetic Agglomeration of Manufacturing and Producer Services in China. J. Quant. Technol. Econ..

[27] Xia Y., Xiong Z. (2021). Technological Diversification, Industry Concentration and Firm Performance Fluctuations. J. Technol. Econ..

[28] Gao L., Pei T., Wang T., Hao Y., Li C., Tian Y., Wang X., Zhang J., Song W., Yang C. (2021). What Type of Industrial Agglomeration Is Beneficial to the Eco-Efficiency of Northwest China?. Sustainability.

[29] Cao N., Niu X., Hu X. (2021). Empirical Study on Financial Support for New Energy Industry Agglomeration Development. Contemp. Econ. Manag..

[30] Eskeland G.S., Harrison A.E. (2003). Moving to greener pastures? Multinationals and the pollution haven hypothesis. J. Dev. Econ..

[31] Zhang J., Wang W., Gao L., Deng Z., Tian Y. (2022). Can the Coal-to-Gas/Electricity policy improve air quality in the Beijing–Tianjin–Hebei region?—Empirical analysis based on the PSM-DID. Atmosphere.

[32] Haque M.A., Biqiong Z., Arshad M.U. (2022). Sources of Financial Development and Their Impact on FDI Inflow: A Panel Data Analysis of Middle-Income Economies. Economies.

[33] Luo J., Zhang Y., Liu X. (2020). Analysis of Characteristics of Meteorological Elements Related to Air Pollution Based on Source Analysis. Environ. Sci. Technol..

[34] Liu M., Fan J., Li Y., Sun L. (2022). Simulating the Spatial Mismatch between Ecosystem Services’ (ESs’) Supply and Demand Based on Their Spatial Transfer in Urban Agglomeration Area, China. Land.

[35] Copeland B.R., Taylor M.S. (1994). North-South Trade and the Environment. Q. J. Econ..

[36] Fu Q., Bao J. (2002). Environmental regulation, FDI and total factor productivity——An example from the Yangtze River Economic Belt. J. Chongqing Technol. Bus. Univ..

[37] Zhao Z., Deng X., Zhang F., Li Z., Shi W., Sun Z., Zhang X. (2022). Scenario analysis of livestock carrying capacity risk in farmland from the perspective of planting and breeding balance in northeast China. Land.

[38] Yang T., Zhu Y. (2021). The Impact of Industrial Co-agglomeration on Sustainable Development of Resource. J. Beijing Inst. Technol..

[39] Han F., Xie Y. (2017). Does the Agglomeration of Producer Services Reduce Carbon Emissions?. J. Quant. Tech. Econ..

[40] Bai Y., Qian Q., Jiao J., Li L., Yang R. (2020). Can Environmental Innovation Benefit from Outward Foreign Direct Investment to Developed Countries? Evidence from Chinese Manufacturing Enterprises. Environ. Sci. Pollut. Res..

[41] Wang H., Ling Y., Luo J. (2022). Impact of Collaborative Agglomeration of Manufacturing and Producer Services on Regional Economic Resilience. J. Ind. Technol. Econ..

[42] Jiang S., Lu C. (2022). Environmental Regulation Affect the Externality and Heterogeneity of Carbon Emission Efficiency: Based on the Analysis of Agglomeration and Synergy of Producer Services. East China Econ. Manag..

[43] Li J., Luo N. (2020). Temporal and Spatial Evolution and Coordination Governance of Haze Pollution in Urban Agglomerations in the Middle Reaches of the Yangtze River from 1998 to 2015. Econ. Geogr..

[44] Zhu Z., Zhu X., Li S. (2021). Evolution process and characteristics of spatial structure of urban agglomeration in the middle reaches of the Yangtze River. Acta Geogr. Sin..

[45] Cao W., Ye P., Zhao H. (2015). Statistics Method of River Density in the Country. Des. Water Resour. Hydroelectr. Eng..

[46] Hashmi R., Alam K. (2019). Dynamic relationship among environmental regulation, innovation, CO_2_ emissions, population, and economic growth in OECD countries: A panel investigation. J. Clean. Prod..

[47] Ehrlich P.R., Holdren J.P. (1971). Impact of population growth. Science.

[48] York R., Rosa E.A., Dietz T. (2003). STIRPAT, IPAT and ImPACT: Analytic tools for unpacking the driving forces of environmental impacts. Ecol. Econ..

[49] Wang X., Wang K., Su L. (2016). Contribution of atmospheric diffusion conditions to the recent improvement in air quality in China. Sci. Rep..

[50] Andreoni J., Levinson A. (2001). The simple analytics of the environmental Kuznets curve. J. Public Econ..

[51] Zhu H.M., Duan L.J., Guo Y.W., Yu K.M. (2016). The effects of FDI, economic growth and energy consumption on carbon emis-sions in ASEAN-5: Evidence from panel quantile regression. Econ. Model..

[52] Doytch N., Narayan S. (2016). Does FDI influence renewable energy consumption? An analysis of sectoral FDI impact on renewable and non-renewable industrial energy consumption. Energy Econ..

[53] Tang C.F., Tan B.W. (2015). The impact of energy consumption, income and foreign direct investment on carbon dioxide emis-sions in Vietnam. Energy.

[54] Liu H., Zhang J. (2022). The Impact of Industrial Cooperative Agglomerationon High-quality Economic Developmen. Sci. Technol. Prog. Policy.

[55] Gao L., Pei T., Zhang J., Tian Y. (2022). The “Pollution Halo” Effect of FDI: Evidence from the Chinese Sichuan–Chongqing Urban Agglomeration. Int. J. Environ. Res. Public Health.

[56] Shad S., Fan M., Yang L. (2013). How does the resource industry dependence affect the economic development. J. Manag. World.

[57] Zou B., Fang X., Feng H., Zhou X. (2019). Simplicity versus accuracy for estimation of the PM_2.5_ concentration: A comparison between LUR and GWR methods across time scales. J. Spat. Sci..

[58] Wu j., Guo Z. (2016). Research on the Convergence of Carbon Dioxide Emissions in China: A Continuous Dynamic Distribution Approach. Stat. Res..

[59] Apergis N., Pinar M., Unlu E. (2022). How do foreign direct investment flows affect carbon emissions in BRICS countries? Revisiting the pollution haven hypothesis using bilateral FDI flows from OECD to BRICS countries. Environ Sci Pollut Res..

[60] He W., Zhang Y. (2021). Environmental Regulation, Industrial Restructuring and High-quality Economic Development: An Analysis Based on PVAR Model of 11 Province. J. Stat. Inf..

[61] Pei T., Gao L., Yang C., Xu C., Tian Y., Song W. (2021). The impact of FDI on urban PM_2.5_ pollution in China: The mediating effect of industrial structure transformation. Int. J. Environ. Res. Public Health.

[62] Guo Q., Wang J., Zhou Z. (2018). Characteristics and reason analysis of a typical heavy air pollution event in Chengdu. Acta Sci. Circumst..

[63] Ma Z., Xiao H. (2017). The research on a spatial differentiation of influence factors of regional PM_2.5_ in China—The empirical analysis based on geographically weighted regression model. J. Shanxi Univ. Financ. Econ..

[64] Wang S., Gao S., Chen J. (2020). Spatial heterogeneity of driving factors of urban haze pollution in China based on GWR model. Geogr. Res..

[65] Washington University, St. Louis, USA. Atmospheric Composition Analysis Group. https://sites.wustl.edu/acag/datasets/surface-pm2-5/.

[66] NOAA Annual Nighttime Stable Light Data from Version 4 DMSP/OLS Nighttime Light Time Series Datasets. https://www.ngdc.noaa.gov/eog/dmsp/downloadV4composites.html.

[67] NOAA Monthly Nighttime Light Data from Version 1 VIIRS Day/Night Band Nighttime Light Datasets. https://www.ngdc.noaa.gov/eog/viirs/download_dnb_composites.html.

[68] National Bureau of Statistics of China (2004–2021). China City Statistical Yearbook.

[69] Statistics Bureau of Jiangxi, NBS Sarvey Office in Jiangxi (2004–2021). Jiangxi Statistical Yearbook.

[70] Statistics Bureau of Hubei, NBS Sarvey Office in Hubei (2004–2021). Hubei Statistical Yearbook.

[71] Statistics Bureau of Hunan, NBS Sarvey Office in Hunan (2004–2021). Hunan Statistical Yearbook.

[72] Tongzhou District People’s Government of Beijing Municipality Industry Classification of the National Economy (GB/T4754-2017). http://www.bjtzh.gov.cn/jxw/fzx/201712/1184715.shtml.

[73] State Statistical Bureau (2019). Statistical Classification of Producer Services. http://www.stats.gov.cn/tjsj/tjbz/201904/t20190417_1660042.html.

[74] Lesage J.P., Pace R.K. (2008). Spatial econometric modeling of origin-destination flows. J. Reg. Sci..

[75] Baron R.M., Kenny D.A. (1986). The moderator-mediator variable distinction in social psychological research: Conceptual, strategic, and statistical considerations. J. Pers. Soc. Psychol..

[76] Zhang Z., Zhang G., Li L. (2022). The Spatial Impact of Atmospheric Environmental Policy on Public Health Based on the Mediation Effect of Air Pollution in China. Environ. Sci. Pollut. Res..

[77] Tobler W.R. (1970). A computer movie simulating urban growth in the detroit region. Econ. Geogr..

